# Genetic and Mitochondrial Metabolic Analyses of an Atypical Form of Leigh Syndrome

**DOI:** 10.3389/fcell.2021.767407

**Published:** 2021-12-22

**Authors:** Martine Uittenbogaard, Kuntal Sen, Matthew Whitehead, Christine A. Brantner, Yue Wang, Lee-Jun Wong, Andrea Gropman, Anne Chiaramello

**Affiliations:** ^1^ Department of Anatomy and Cell Biology, George Washington University School of Medicine and Health Sciences, Washington, DC, United States; ^2^ Children’s National Medical Center, Division of Neurogenetics and Developmental Pediatrics, Washington, DC, United States; ^3^ Children’s National Medical Center, Division of Radiology, Washington, DC, United States; ^4^ GW Nanofabrication and Imaging Center, Office of the Vice President for Research, George Washington University, Washington, DC, United States; ^5^ Department of Molecular and Human Genetics, Baylor College of Medicine, Houston, TX, United States

**Keywords:** leigh syndrome, whole exome sequencing, combined oxidative phosphorylation deficiency, mitochondrial energy metabolism, metabolic adaptability, nuclear variants

## Abstract

In this study, we aimed to establish the mitochondrial etiology of the proband’s progressive neurodegenerative disease suggestive of an atypical Leigh syndrome, by determining the proband’s pathogenic variants. Brain MRI showed a constellation of multifocal temporally disparate lesions in the cerebral deep gray nuclei, brainstem, cerebellum, spinal cord along with rhombencephalic atrophy, and optic nerve atrophy. Single voxel ^1^H MRS performed concurrently over the left cerebral deep gray nuclei showed a small lactate peak, increased glutamate and citrate elevation, elevating suspicion of a mitochondrial etiology. Whole exome sequencing revealed three heterozygous nuclear variants mapping in three distinct genes known to cause Leigh syndrome. Our mitochondrial bioenergetic investigations revealed an impaired mitochondrial energy metabolism. The proband’s overall ATP deficit is further intensified by an ineffective metabolic reprogramming between oxidative phosphorylation and glycolysis. The deficient metabolic adaptability and global energy deficit correlate with the proband’s neurological symptoms congruent with an atypical Leigh syndrome. In conclusion, our study provides much needed insights to support the development of molecular diagnostic and therapeutic strategies for atypical Leigh syndrome.

## Introduction

Leigh syndrome (LS; OMIM No. 256000), originally described in 1951 by the British neuropathologist Dr. Dennis Leigh, is a subacute necrotizing encephalomyelopathy with a frequency gliosis in several brain regions (Leigh, 1951). It affects about 1 in 40,000 live births and can caused by more than 75 distinct genes (Lake et al., 2016). Onset of symptoms usually begins between 3 months and 2 years of age and rarely during adolescence or early adulthood. The diagnostic criteria for this intractable progressive neurodegenerative disease includes symmetrical brain lesions in the brainstem or cerebral deep gray nuclei, elevated lactate in the cerebrospinal fluid or brain, and clinical findings suggestive of a mitochondrial disease (Ruhoy and Saneto, 2014; Whitehead et al., 2016). Therefore, MRI and MRS are important for diagnosis and disease monitoring. LS patients exhibit an extensive clinical heterogeneity that makes the clinical diagnosis of LS challenging. The most frequent clinical symptoms include developmental delay and regression, seizures, ataxia, dystonia, ophthalmoparesis, optic atrophy, sensorineural hearing loss, dysphasia, failure to thrive, and respiratory problems (Finsterer, 2008; Chang et al., 2020). LS clinical heterogeneity can result in Leigh-like syndrome or atypical Leigh syndrome in patients with atypical neuropathology and clinical presentation with symptoms affecting the peripheral nervous system, including polyneuropathy and myopathy, and non-neurological symptoms, such as cardiomyopathy, renal failure, short stature, anemia, diabetes, and gastrointestinal dysfunctions (Finsterer, 2008).

Most patients with LS suffer from a neurometabolic crisis during childhood that is often associated with a febrile illness. This impaired energy generation is caused by a dysfunctional oxidative phosphorylation (OXPHOS) pathway, which makes mitochondrial ATP *via* four respiratory complexes, Complex I to IV, and ATP synthase (also called Complex V). LS is predominantly monogenic with more than 75 identified pathogenic nuclear and mitochondrial variants relevant to its molecular pathogenesis (Lake et al., 2016). Most of them map in mitochondrial or nuclear genes encoding subunits of the respiratory complexes or proteins required for their assembly, stability and activity. These pathogenic variants result in dysfunctional Complexes I, III, IV, or V, as well as coenzyme Q10 deficiency or pyruvate dehydrogenase complex deficiencies (Finsterer, 2008). Despite the recent remarkable genetic advances with the advent of next-generation sequencing (NGS), the pathogenic mechanisms of LS remain elusive given its highly heterogenous genetic etiology that obscures the genotype-phenotype correlation. Currently, many patients clinically diagnosed with LS or Leigh-like syndrome remain without a genetic diagnosis, suggesting that additional pathogenic causative variants or a combination of pathogenic variants remain to be discovered.

Here, we report the case of 6-year-old proband with neurological manifestations suggestive of an atypical form of LS harboring three heterozygous variants mapping in three distinct genes known to cause LS. Our comprehensive functional investigations of the proband’s mitochondrial energy metabolism confirm the suspected mitochondrial etiology.

## Methods

### Ethical Issues

This study was approved by the Institutional Review Board of the George Washington University and Children’s National Medical Center and was conducted in accordance with the ethical principles of the Declaration of Helsinki of 1975 (revised 1983). Patient skin biopsy was performed after receiving written informed consent from the legally authorized representatives (parents of the proband) with permission to study the derived dermal fibroblasts.

### Skin Biopsy

A 3 mm skin biopsy was performed on the 5-year-old proband, from which dermal fibroblasts were derived as described (Uittenbogaard et al., 2018a). Derived fibroblasts were frozen at passage two and never used beyond passage 10. Human primary dermal fibroblasts from a healthy subject (Cat# GM03377E) were obtained from the Coriell Cell Repositories (Camden, NJ).

### Transmission Electron Microscopy

Fibroblasts from the proband and a control subject were fixed in 2.5% glutaraldehyde, 1% paraformaldehyde in 0.12 M sodium cacodylate buffer as described (Uittenbogaard et al., 2018b). Samples were imaged with a FEI Talos F200X-transmission electron microscope (Thermo Fisher).

### Genetic Testing

Total genomic DNA was isolated from blood samples from the proband to perform WES and LR-PCR-MPS of the mitochondrial genome by the Medical Genetics Laboratories at Baylor College of Medicine, as described (Cui et al., 2013; Uittenbogaard et al., 2019). Reads were aligned to the human reference genome (UCSC hg19) using the NextGEN software (SoftGenetics; State College, PA). Variants were identified and annotated using an in-house bioinformatic pipeline with our filtering strategy summarized in Figure 2 of Uittenbogaard et al. (2019). The pathogenicity of variants was evaluated using the American College of Medical Genetics and Genomics guidelines by board-certified molecular geneticists. Computational analysis of nuclear variant’s pathogenicity was performed using PolyPhen-2 and SIFT.

### Live-Cell Measurements of Mitochondrial Respiratory and Glycolytic Activity

The bioenergetic status of the proband’s fibroblasts was measured using the Seahorse Extracellular Flux XFp Analyzer (Agilent Technologies; Santa Clara, CA), as described (Gropman et al., 2020). Optimal cell density (5,000/well) and the concentration of FCCP (fluoro 3-carbonyl cyanide-methoxyphenyl hydrazine; 2 µM) were determined using the Cell Energy Phenotype Test kit. Using the XFp Mito Stress Test kit, oxygen consumption rate (OCR) and extracellular acidification rate (ECAR) were measured under basal conditions and after sequential injections of oligomycin (1 µM), FCCP (2 µM), and a mix of rotenone and antimycin A (1 µM). Using the Seahorse XFp real-time ATP Rate assay, we simultaneously quantified the rate of ATP produced by OXPHOS and glycolysis according to the manufacturer’s recommendations. OCR and ECAR were measured under basal conditions and after sequential injections of oligomycin (1.5 µM) and a mix of rotenone and antimycin A (0.5 µM). Using the XFp Glycolytic Rate Assay, we analyzed the glycolytic rate by quantifying the total proton efflux and the glycolytic proton efflux as described (Uittenbogaard et al., 2018a).

All the data from three independent experiments, each including three technical replicates, were normalized to cell numbers after the assay and plotted as OCR (pmol/min/cell ± S.E.M.), and ECAR (mpH/min/cell ± S.E.M.) using the Seahorse MultiReport Generator software. Statistical analyses were performed using the unpaired student t-test with *p*-value of less than 0.05 considered statistically significant.

## Results

### Clinical History

The 6-year-old male proband has an unclear clinical diagnosis exhibiting developmental delay, hypotonia, spasticity, ptosis, bilateral cataracts and sensorineural hearing loss. His global symptomatology is consistent with a suspected inborn error of metabolism of uncertain etiology. The proband’s parents, a 46-year-old father and a 39-year-old mother, are of African-American descent and nonconsanguineous. Maternal history was significant with five miscarriages in the setting of hypercoagulability due to a factor 2 mutation for which she is treated with Lovenox. She had gestational diabetes that was treated with diet.

The proband was born at 35 weeks’ gestation by C-section due to fetal deceleration and weighed 2.4 kg. During the neonatal period, he had hypoglycemia, requiring admission to the intensive care unit. He passed his newborn hearing screen and was discharged 1 week after delivery. At 5 months, a brain CT was performed to investigate anterior fontanelle bulging, which showed enlarged subarachnoid spaces, an absent thalamic massa intermedia, and a residual cavum septum pellucidum/vergae, but otherwise, normal brain density, texture, and volume. At 6 months, the proband had his first ophthalmology evaluation due to concerns for corneal opacities. He was diagnosed with bilateral infantile cataracts and congenital ptosis of the right upper lid. He underwent a surgical procedure for extraction of his right cataract and a sling for his right upper lid. He was diagnosed with plagiocephaly and hospitalized due to a bulging fontanelle and irritability. Due to his staring episodes, an electroencephalogram was performed, which failed to show any epileptiform discharges.

**FIGURE 1:**
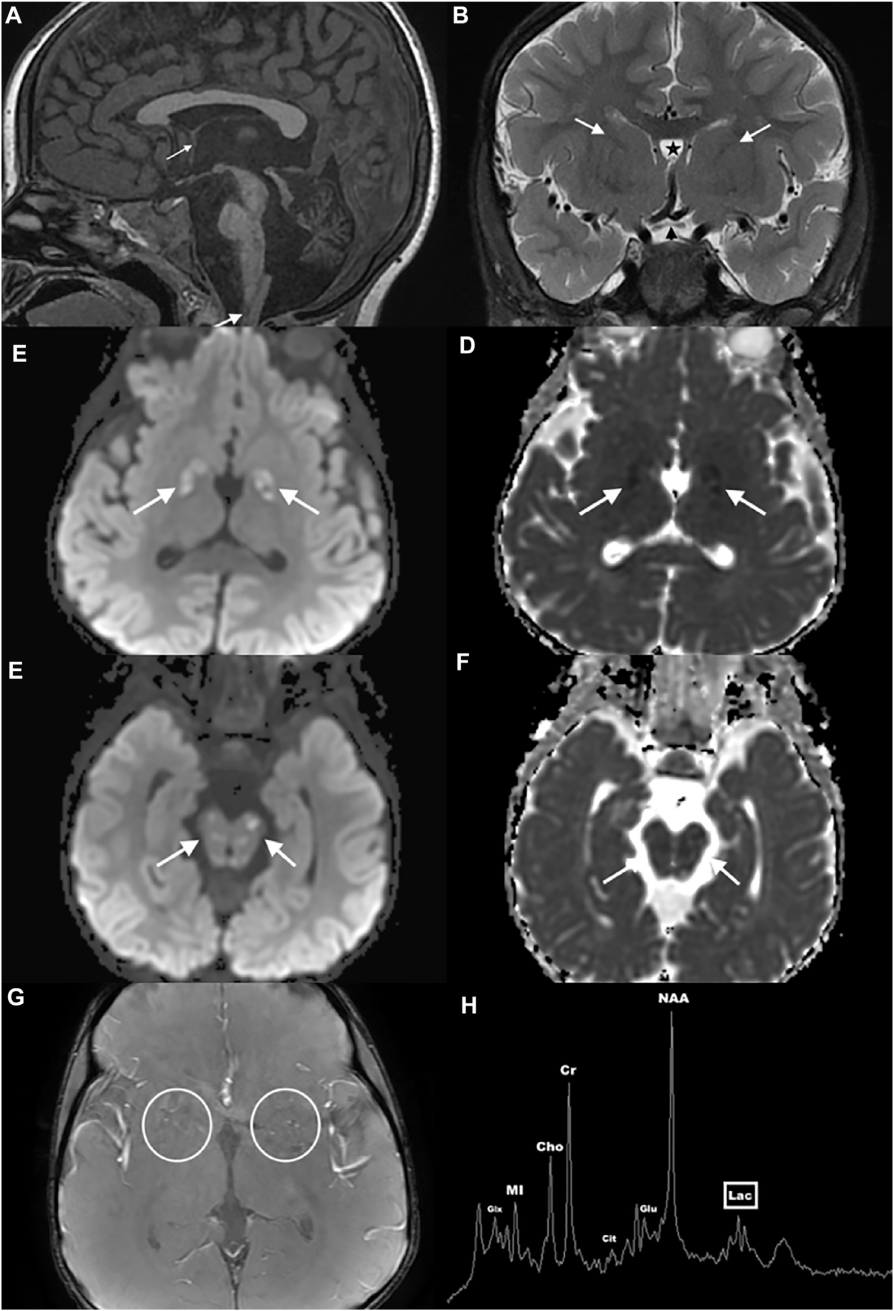
Brain MRI and MRS of the proband. Sagittal midline T1WI **(A)** shows atrophy of the cerebellum and brainstem, hypoplasia of the anterior commissure (thin arrow), absence of the thalamic massa intermedia, and multiple hypointense lesions in the brainstem, cerebellum, and cervical spinal cord (thick arrow). Coronal T2WI **(B)** reveals optic pathway hypoplasia (black arrowhead), deficiency of the anterior limbs of the internal capsules (white arrows), and a thick-walled cavum septum pellucidum/vergae (star). Axial susceptibility-weighted angiography image **(C)** depicts pronounced small vessel hypervascularity (circles). Axial diffusion weighted images through the globus pallidus **(D,E)** and midbrain **(F,G)** show multifocal varying aged lesions with mixed facilitated and reduced diffusion consistent with acute on chronic metabolic injury (arrows). Single voxel ^1^H MRS over the left cerebral deep gray nuclei **(H)** shows a small lactate peak (lac), increased glutamate (glu), and citrate (cit) elevation.

At 16 months, he showed signs of developmental delay with mild hypotonia and delayed walking. He was referred to physical and speech therapists and diagnosed with sensorineural hearing loss. A temporal bone CT performed at 30 months showed normal appearing inner ear structures, but disclosed interval cerebellar atrophy. A brain MRI at 32 months confirmed cerebellar atrophy and absent massa intermedia (Figures 1A–G). It also revealed brainstem atrophy, hypoplasia of the anterior commissure, optic pathway, and olfactory system as well as cerebral malformations including abnormal parietoinsular gyration, a thick-walled cavum septum pellucidum/vergae, and deficiency of the anterior limb of the internal capsule. Furthermore, multifocal lesions of varying ages were present with diffusion abnormalities ranging from reduced to facilitated, involving the globi pallidi, midbrain (including but not limited to the periaqueductal gray matter, colliculi, and substantia nigra), pontine tegmentum, medulla, cerebellar gray and white matter, and imaged cervical spinal cord consistent with acute on chronic metabolic injury. Many of the small vessels within and along the brain surface were prominent and consistent with hypervascularity, especially in the basal ganglia. These findings were again demonstrated when MRIs were done at the age of five and six. Single voxel ^1^H MRS performed concurrently over the left cerebral deep gray nuclei showed a small lactate peak, increased glutamate, and citrate elevation, elevating suspicion of a mitochondrial etiology. The proband was referred to our Neurogenetics clinic upon suspicion of a neurometabolic disease with a mitochondrial etiology based on his significant developmental regression and several lactic acidosis episodes.

At 5 years, he had a facial droop and underwent another brain MRI that showed recurrent small vessel hypervascularity and additional acute on chronic lesions in similar anatomic regions of the basal ganglia, brainstem, and cerebellum, consistent with metabolic disease progression. A single voxel ^1^H MRS over the left cerebral deep gray nuclei confirmed mild lactate, increased glutamate, and citrate elevation (Figure 1H). The proband was treated with intravenous dextrose during episodes of metabolic decompensation and started on levocarnitine at three doses of 330 mg. Although the proband’s levels of alpha-aminoadipate and cystathionine were elevated, his acylcarnitine and very long chain fatty acid levels were normal. His screen for congenital disorder of glycosylation was negative. EKG showed normal sinus rhythm, and echocardiography showed no structural abnormalities. Following several episodes of choking, the proband underwent a swallow evaluation, which revealed prolonged oral phase of the swallow with no aspiration of solid food.

At 6 years, examination revealed dysmorphic features including bilateral ptosis, pendulous lips, small chin, and myopathic facies. His bilateral cataracts reduces his visual acuity. He only perceives movements. He has some axial hypotonia with bilateral spasticity in lower extremity. His walking is severely limited, being able to only take a few steps in his gait trainer without assistance.

The constellation of clinical features and brain MRI findings of the proband are consistent with an LS-like phenotype. He exhibits additional atypical features, including the clinical findings of dysmorphism, the neuroimaging findings of absent thalamic massa intermedia, residual cavum septum pellucidum/vergae, hypoplasia of the anterior commissure, optic pathway and olfactory system, as well as cerebral malformations including abnormal parietoinsular gyration, a thick-walled cavum septum pellucidum/vergae, and deficiency of the anterior limb of the internal capsule.

### Genetic Diagnosis

The proband’s mitochondrial genome, which was sequenced by LR-PCR-MPS, does not harbor any pathogenic mitochondrial variants. WES was performed using blood sample from the proband applying the filering strategy as described in Figure 2 of Uittenbogaard et al. (2019). After filtering steps were applied to 30,000 variants, we removed artifacts and false positive variants, and performed our analysis on the 25,000 remaining variants that revealed the presence of three heterozygous nuclear variants in the *EARS2*, *MTFMT*, and *NARS2* genes, all linked to combined oxidative phosphorylation deficiency (COXPD) (Table 1). The *EARS2* c.368T > A (p.L123Q) variant maps in the exon three of the EARS2 protein, which regulates mitochondrial protein translation by attaching glutamate to the cognate mitochondrial t-RNA (Mai et al., 2017). It is reported at a low allele frequency in the population databases dbSNP (www.ncbi.nlm.nih.gov/snp/) and gnomAD (http://gnomad.broadinstitute.org/variant). Both the SIFT and PolyPhen2 algorithms predicted the p. L123Q variant to be deleterious. Exon 3, which encodes the one of the four catalytic domains of mitochondrial glutamyl-tRNA synthetase, is a hotspot for nuclear variants (Steenweg et al., 2012). Pathogenic EARS2 variants are associated with the rare mitochondrial disease COXPD 12 (OMIM No. 614924) (Talim et al., 2013). The novel *MTFMT* variant c.20G > A (p.R7H) is predicted pathogenic and maps in exon 1 of the mitochondrial methionyl-tRNA formyltransferase (MTFMT) protein (Table 1). Several pathogenic *MTFMT* variants are associated with COXPD 15 (OMIM No 614947) (Neeve et al., 2013). The pathogenic *NARS2* variant c.791C > G (p.S264C) maps in the exon 7 that in part encodes the catalytic domain of the mitochondrial asparaginyl-tRNA synthetase 2 (Table 1). Several *NARS2* variants are linked to COXPD 24 (OMIM No. 616239) (Simon et al., 2015; Valander et al., 2015; Seaver et al., 2018).

**FIGURE 2:**
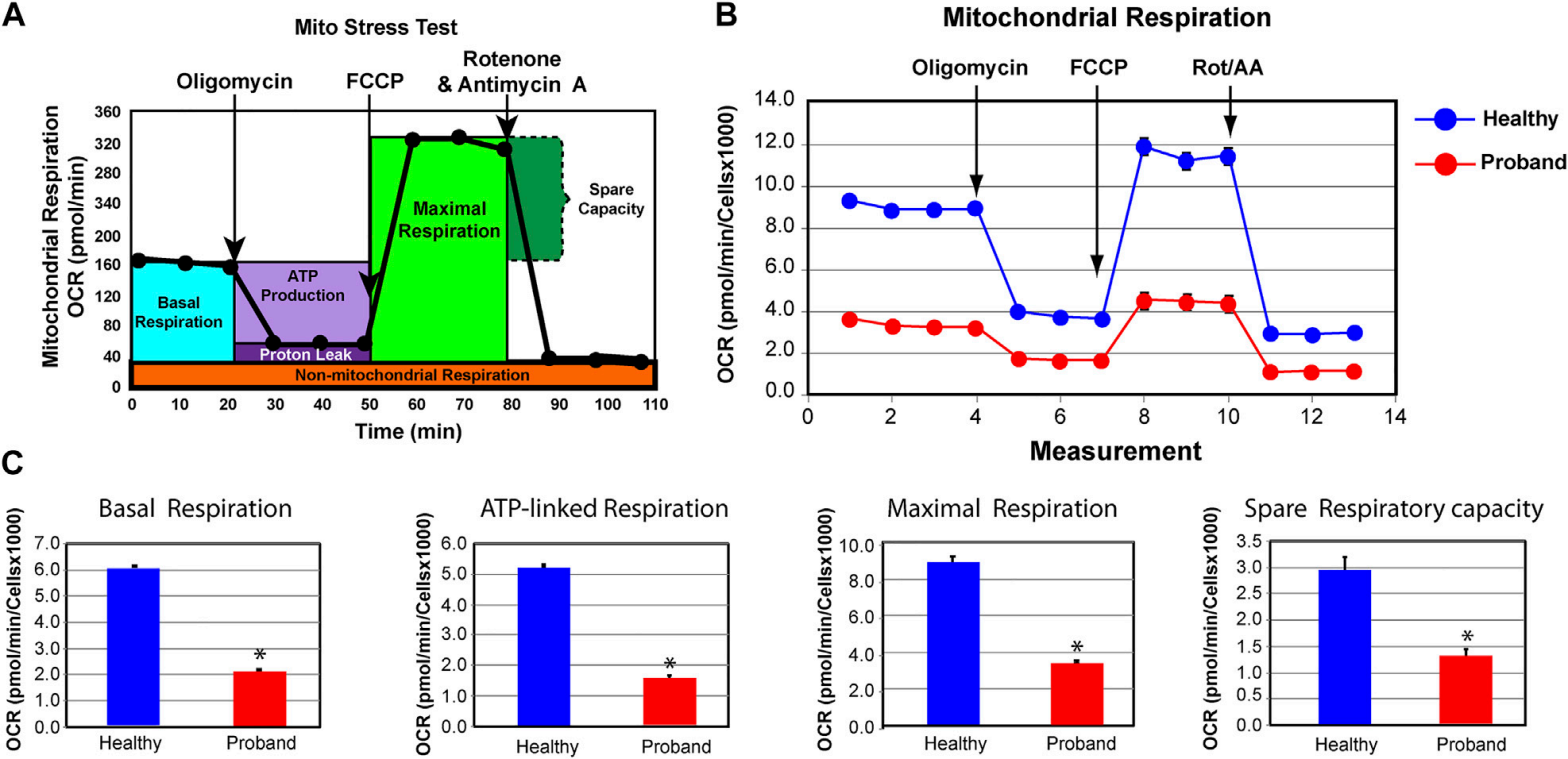
The proband’s fibroblasts exhibit a deficient mitochondrial bioenergetic capacity. **(A)** Profile of the oxygen consumption rate (OCR) adapted from the Agilent Technologies brochure of the Mitochondrial Stress Test. **(B)** OCR profiles of the proband (red) and healthy subject (blue). **(C)** Quantitative data of basal respiration, ATP-linked respiration, maximal respiration, and spare respiratory capacity. The healthy subject is shown in blue while the proband is illustrated in red. Data are represented as means ± S.E.M., *n* = 3 of independent experiments, each with three technical replicates. * indicates statistically significant differences with a *p* value of 0.0001.

**TABLE 1:** Nuclear variants of the proband’s fibroblasts revealed by whole exome sequencing.

Gene	Inheritance pattern	OMIM	Disease	Nucleotide	Variant	Location	Zygosity	Reference	Classification
EARS2	AR	612799	coxpd12	c.368T > A	p.L123Q	Exon 3	Het	rs968976447	VUS
MTFMT	AR	611766	coxpd15	c.20G > A	p.R7H	Exon 1	Het	Not reported	VUS
NARS2	AR	612803	coxpd24	c.791C > G	p.S264C	Exon 7	Het	rs141507678	Pathogenic

Abbreviations: AR, autosomal recessive; Coxpd, combined oxidative phosphorylation deficiency; Het, heterozygous; VUS, variant of unknown significance.

### Functional Studies of the ATP Metabolism

Based on the presence of these variants, we investigated the ATP metabolism using the proband’s fibroblasts from a skin biopsy performed at the age of five. As a control subject, we used commercially available dermal fibroblasts from a healthy subject of similar age range whose metabolic profile has already been characterized and comparable to two other healthy subjects (Uittenbogaard et al., 2018b). We measured OCR, a key functional indicator of the mitochondrial energy metabolism, to accurately assess OXPHOS parameters using the Mitochondrial Stress Test assay. The proband’s OXPHOS parameters were greatly reduced, when compared to those of a healthy subject (Figure 2). We found a 65% decline of the basal respiration and a 69% reduction of ATP-linked respiration (Figure 2C). The maximal respiratory capacity evoked by the protonophore FCCP dropped by 62% (Figure 2C). Finally, the spare respiratory capacity decreased by 55%, hindering the ability to sustain an energy crisis (Figure 2C).

**FIGURE 3:**
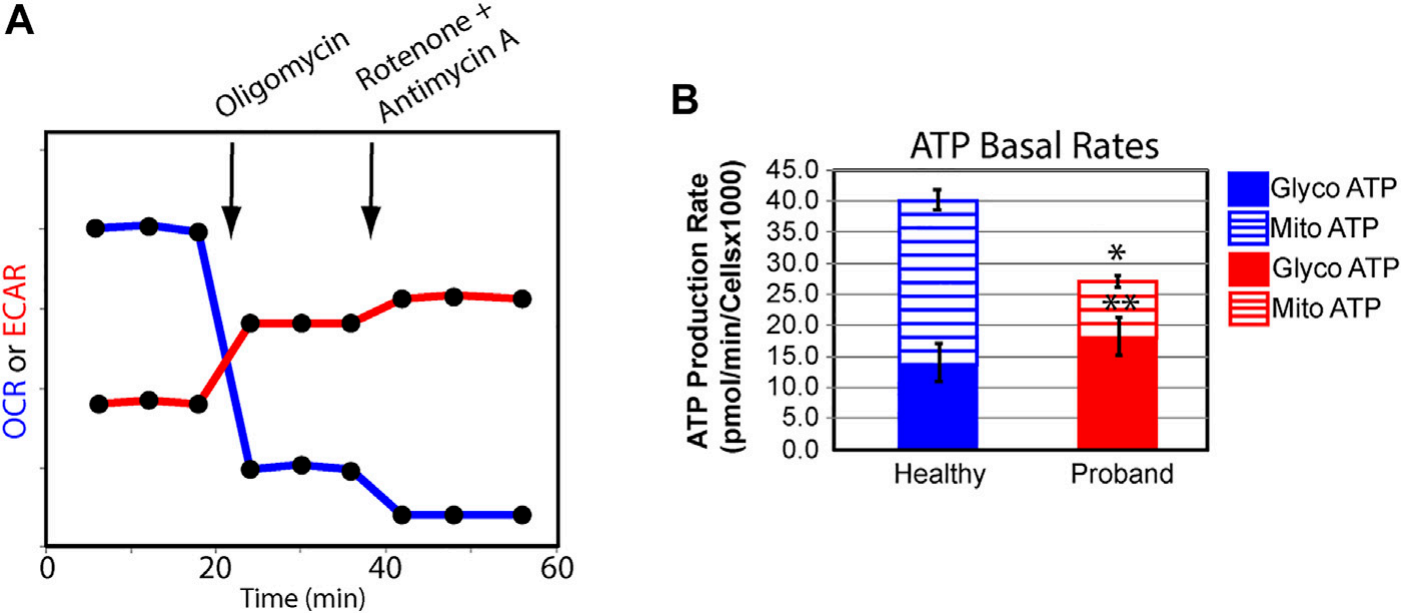
The proband’s fibroblasts display a deficit in total ATP rate production. **(A)** Schematic representation of the Agilent Seahorse XFp Real-Time ATP rate assay during which both OCR and ECAR are simultaneously measured upon compound injections of mitochondrial inhibitors as indicated on the graph. **(B)** Quantification of ATP basal rate from glycolysis (blue for the healthy subject and red for the proband) and mitochondrial OXPHOS (blue hatched column for the healthy subject and red hatched column for the proband). Data are represented as means ± S. EM., *n* = 3 of independent experiments, each with three technical replicates. * and ** indicate a *p* value of 0.001 and 0.05, respectively.

We next examined the rate of ATP production in the proband’s fibroblasts from glycolysis and OXPHOS using the XFp Real-Time ATP rate assay. Both OCR and ECAR were simultaneously measured upon injection of oligomycin followed by injection of rotenone and antimycin A to fully inhibit mitochondrial ATP production (Figure 3A). The proband’s mitochondrial rate of ATP production decreased by 63% (Figure 3B), corrobating the overall OXPHOS decline. The 16% increase in glycolysis-mediated ATP production rate was not enough to offset the 31% decrease in total ATP rate production.

**FIGURE 4:**
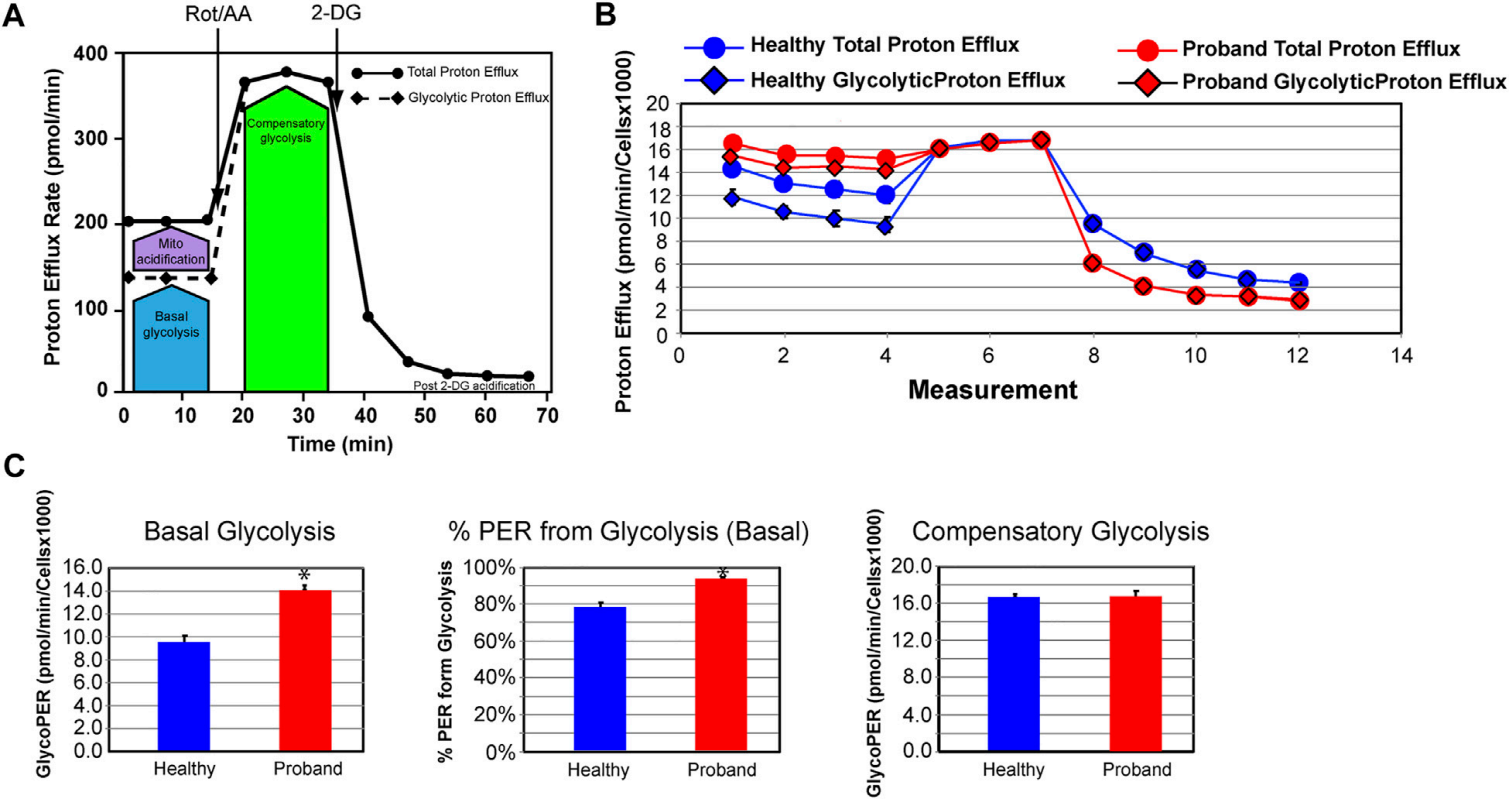
The proband’s fibroblasts do not possess an increased compensatory glycolysis to compensate for the OXPHOS deficit. **(A)** Schematic representation of the Agilent Seahorse XFp Glycolytic Rate assay adapted from the Agilent Technologies brochure. **(B)** Profiles of the proton efflux rate (PER) of the proband (red) and a healthy subject (blue). **(C)** Quantitative analysis of three key bioenergetic markers for glycolysis: basal glycolysis, %PER from basal glycolysis, and compensatory glycolysis (healthy subject in blue and proband in red). Data are represented as means ± S.E.M., *n* = 3 of independent experiments, each with three technical replicates. * indicates statistically significant differences with a *p* value of 0.0001.

We next investigated the glycolytic metabolism of the proband using the Glycolysis Rate assay, which accurately assesses glycolytic activity by correlating one-to-one with lactate accumulation. The total Proton Efflux Rate (PER) and the Glycolytic Proton Efflux Rate (GlycoPER) were measured using both OCR and ECAR values to account for mitochondrial (CO_2_) acidification from the mitochondrial TCA cycle (Figures 4A,B) (Mookerjee et al., 2017). Basal glycolysis increased by 34%, which was confirmed by an increased PER to 94%, compared to 79% in healthy fibroblasts (Figure 4C). We measured the compensatory glycolysis response as an indication of metabolic reprogramming toward glycolysis following a provoked energy crisis by rotenone and antimycin A. The proband’s fibroblasts failed to increase glycolysis as a mean to compensate for this acute mitochondrial ATP crisis (Figure 4C).

### Mitochondrial Morphometric Analysis

**FIGURE 5:**
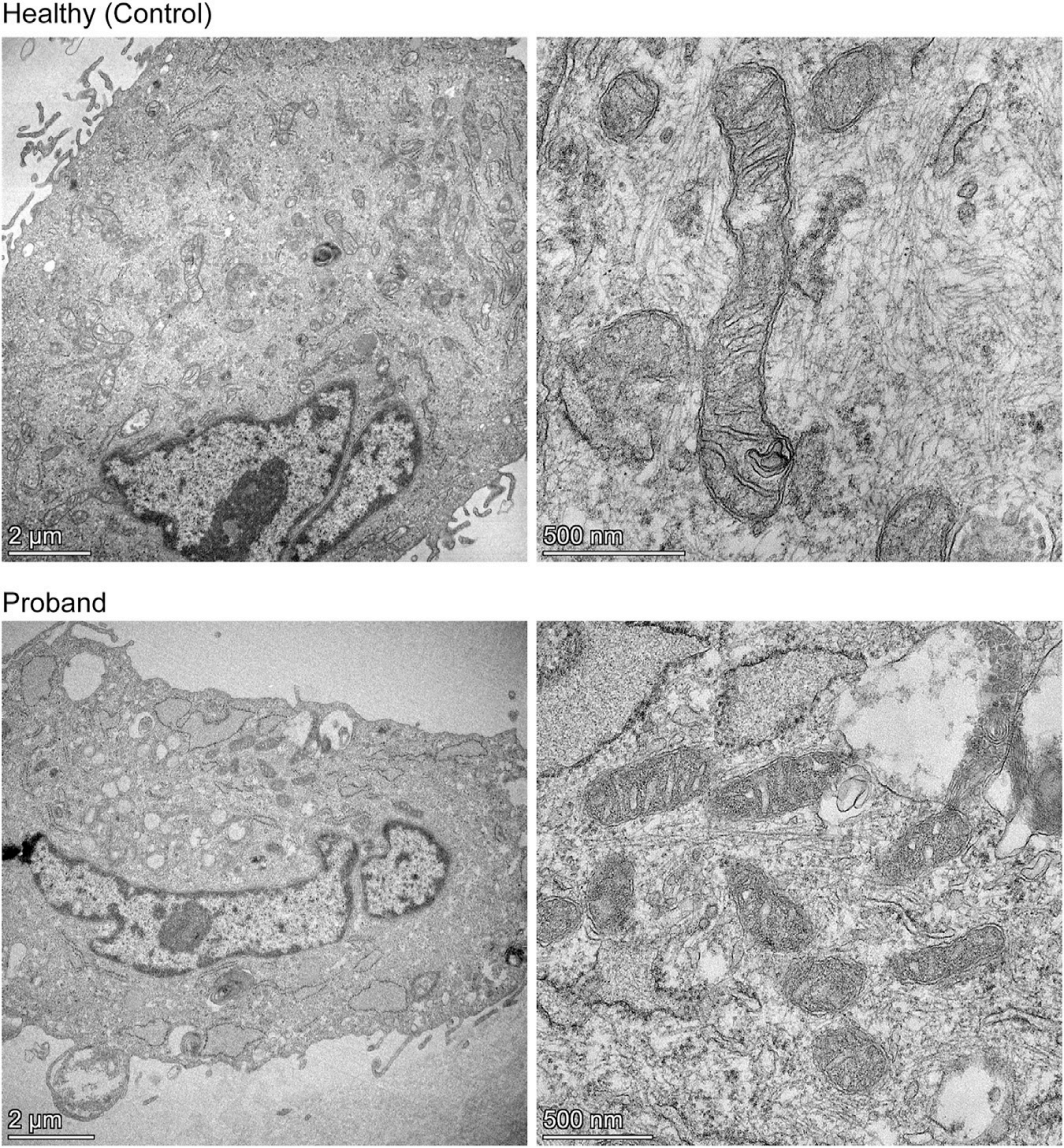
Mitochondrial morphometric analysis by transmission electron microscopy. Representative electron micrographs of healthy dermal fibroblasts **(top)** and the proband’s dermal fibroblasts **(bottom)** are shown. The left panels illustrate the decreased mitochondrial population in the proband **(bottom)** compared to that of a healthy subject **(top)** (scale bar = 2 µm). The right panels show high magnification of mitochondria from a healthy subject **(top)** or proband **(bottom)**, highlighting the difference in length, and cristae morphology (scale bar = 500 nm).

We then conducted a mitochondrial morphometric analysis using transmission electron microscopy to investigate whether the proband’s mitochondria showed ultrastructural defects. The proband’s fibroblasts contained a decreased mitochondrial population, when compared to that of healthy fibroblasts (Figure 5). The proband’s mitochondria were small with rare and swollen cristae (Figure 5). These morphometric results confirm the proband’s dysfunctional mitochondrial energy metabolism (Figures 2–4).

## Discussion

Our study reports a young proband with a constellation of multifocal temporally disparate lesions in the cerebral deep gray nuclei, brainstem, cerebellum, and spinal cord along with rhombencephalic atrophy, optic nerve atrophy, and brain lactate that is highly suggestive of LS (Gonçalves et al., 2020). Bilateral lesions in the basal ganglia, thalamus (mostly medially), brainstem (substantia nigra, oculomotor nuclei, periaqueductal gray matter), pontine tegmentum, and inferior olivary nuclei are the most common imaging findings in LS (Gonçalves et al., 2020). Cerebellar and spinal cord lesions may also be found. Leigh syndrome–related neuropathologic changes include varying aged vasculonecrotic lesions in the deep gray nuclei and brainstem. Acute edematous lesions evolve to gliosis with hypervascularity, and ultimately, chronic infarcts with necrosis and gliosis (Gonçalves et al., 2020). Active brain lesions demonstrate hyperperfusion, possibly from lactic acidemia-induced vasodilation and lesional perfusion superimposed on small-vessel proliferation, which accounts for the hypervascularity seen in the proband^4^. Such hyperperfusion may herald disease progression (Whitehead et al., 2016). Structural brain abnormalities are unusual in patients with inborn metabolic errors. Energetic disorders, such as inherited mitochondrial diseases, are an exception, since neuronal formation, proliferation, migration, and organization require adequate energy production (Whitehead et al., 2015). This is congruent with the patient’s cerebral malformations.

Our WES analysis reveals three heterozygous nuclear variants, *MTFMT*, *NARS2*, and *EARS2*, mapping in genes known to cause COXPD associated with Leigh syndrome (Eichers et al., 2004; Tucker et al., 2011; Neeve et al., 2013; Simon et al., 2015; Lake et al., 2016). The novel *MTFMT* variant with a predicted pathogenicity revealed by our WES analysis adds further genetic heterogeneity to LS (Hayhurst et al., 2019). The most common *MTFMT* pathogenic variant, c.626C > T, was first reported to cause LS (Tucker et al., 2011) given its role during initiation of mitochondrial protein translation (Haack et al., 2014). The pathogenic *NARS2* variant (p.S264C) maps in the catalytic domain of the mitochondrial asparaginyl-tRNA synthase protein, which catalyzes the binding of asparagine to its cognate mt-tRNA (Bonnefond et al., 2005). Several studies reported *NARS2* variants in LS patients (Simon et al., 2015; Sofou et al., 2015; Mizuguchi et al., 2017; Lee et al., 2020; Sofou et al., 2021). More relevant is the sensorineural hearing impairment, as a hallmark of *NARS2*-associated LS phenotype, which is consistent with the proband’s symptoms (Sofou et al., 2021). Additional NARS2-linked phenotype includes intellectual disability, epilepsy, and severe myopathy, all exhibited by the proband (Simon et al., 2015). The proband harbors the *EARS2* c.368T > A (p.L123Q) variant, predicted to be pathogenic by computer-based algorithms, expands the genotyping spectrum of LS-like syndrome. It maps in the exon 3, a hot spot for variants causing severe infantile neurological disorders affecting the white matter with high lactate levels (Steenweg et al., 2012; Talim et al., 2013). Talim et al., 2013 reported an *EARS2*-driven clinical spectrum overlapping with the proband’s symptoms: mother with gestational diabetes, incomplete cleft palate, myopathy, hypotonia, and lactic acidosis in the neonatal period.

Our bioenergetic analysis confirms dysregulated OXPHOS pathway and deficient mitochondrial ATP rate. The decrease in basal and ComplexV-driven respiration is congruent with the reported COXPD caused by pathogenic *MTFMT*, *NARS2*, and *EARS2* variants (Boczonadi et al., 2018). More notably, is the proband’s deficit in the spare respiratory capacity that hinders the ability to avert bioenergetic exhaustion (Brand and Nicholls, 2011; Nielsen et al., 2017). A deficient spare respiratory capacity in skeletal muscle cells leads to reduced physical activities, exercise intolerance, eventually to sarcopenia (Hiona et al., 2010). Since firing neurons require 80% of the spare respiratory capacity (Nicholls, 2002), such deficit results in mental exhaustion and developmental regression. Dysregulated spare respiratory capacity has been reported in patients with other neurodevelopmental mitochondrial diseases (Oláhová et al., 2015; Uittenbogaard et al., 2018a; Uittenbogaard et al., 2018b; Uittenbogaard et al., 2019; Gropman et al., 2020). The proband’s ATP deficit is further intensified by an ineffective metabolic reprogramming from OXPHOS to glycolysis.

Our results on the patient-derived fibroblasts’ mitochondrial energy deficit intersect with our TEM-based mitochondrial morphometric analyses revealing decreased mitochondrial population and altered ultrastructural morphology of cristae. The patient’s cristae are poorly developed and swollen, congruent with the deficient OXPHOS pathway demonstrated by mitochondrial live-cell respiratory assays. Several studies have provided evidence that the cristae shape determines the assembly and stability of respiratory chain complexes, as the cristae house the OXPHOS machinery (Cogliati and Frezza 2013; Cogliati et al., 2016; Aflzal et al., 2021). Our TEM findings of decreased mitochondrial population suggest altered mitochondrial biogenesis and/or mitochondrial dynamics, both processes requiring bioenergetically competent mitochondria (Baxter et al., 2012; Uittenbogaard and Chiaramello, 2014).

In sum, the deficient metabolic adaptability and global energy deficit correlate with the proband’s neurological symptoms and confirm the suspected mitochondrial etiology. Our mitochondrial metabolic and morphometric analyses lend credence to the three heterozygous variants, *MTFMT*, *NARS2*, and *EARS2*, mapping in genes linked to COXPD associated with Leigh syndrome, as a probable cause of the proband’s neurological manifestations and mitochondrial etiology. Recently, oligogenic inheritance of heterozygous variants has increasingly been recognized as a pathogenic mechanism underlying complex phenotypes of metabolic myopathies and mitochondrial neurodegenerative diseases with a deficit in energy metabolism (Khoda and Tokuzawa 2016; Pang and Teo 2017; Sambuughin et al., 2018; Stenton and Prokisch, 2020; Costantini et al., 2021). Thus, our study extends the traditional approach of a single-gene disorder linked to mitochondrial inborn errors of metabolism diseases with the concept of multiple heterozygous variants mapping in genes known to cause monogenic metabolic disorders. The advent of next-generation sequencing-based diagnostics and the burgeoning era of personalized medicine will most likely contribute to the pathogenic complexity of mitochondrial disorders, thereby improving the public genotype-phenotype databases.

## Data Availability

The raw data supporting the conclusion of this article will be made available by the authors, without undue reservation.

## Abbreviations

COXPD, combined oxidative phosphorylation deficiency; DMEM, dulbecco’s modified eagle medium; ECAR, extracellular acidification rate; EEG, electroencephalogram; FCCP, fluoro 3-carbonyl cyanide-methoxyphenyl hydrazine; FGF-2, fibroblast growth factor-2; GlycoPER, glycolytic proton efflux rate; HEPES, 4-(2-hydroxyethyl)-1-piperazineethanesulfonic acid; LR-PCR-MPS, long range PCR followed by massively parallel sequencing; LS, leigh syndrome; MRP, mitochondrial ribosomal protein; MRS, magnetic resonance spectroscopy; MTFMT, mitochondrial methionyl-tRNA formyltransferase; NGS, next-generation sequencing; OCR, oxygen consumption rate; OXPHOS, oxidative phosphorylation; PER, proton exchange rate; SNP, single nucleotide polymorphism; VUS, variant of unknown significance; WES, whole exome sequencing.
